# Supplementary material for: Integrative multi‐omics analysis of intestinal organoid differentiation
Source: Mol Syst Biol. 2018 Jun 26;14(6):e8227. doi: 10.15252/msb.20188227 (PMC6018986; doi:10.15252/msb.20188227)
Supplement: Supplementary file 1 — Expanded View Figures PDF [file MSB-14-e8227-s001.pdf]

Expanded View Figures

Figure EV1. Validation cell-type-enriched culturing methods, metabolome dynamics, and mitochondria abundance and bioenergetics in differentiating organoids.

A GSEA of Lgr^{+} and Lgr^{-} intestinal cell signatures from Munoz *et al* (2012) for CV- and EN-enriched proteins.
B Heatmap showing the relative abundances of significantly changing metabolites measured with mass spectrometry-based lipidomics and metabolomics. Relative change in abundance is shown as the $\log_2$ fold change over the row mean. Different classes of metabolites are color-coded. Significantly enriched Reactome pathways ($P < 0.05$) are shown next to the corresponding cluster of metabolites.
C Correlation heatmap of two independent proteomics datasets of cell-type-enriched organoids cultures. The different genetic background organoids lack the GFP reporter compared to the Lgr5-GFP-DTR organoids.
D Overview of basal ECAR (glycolysis) and OCR (mitochondrial respiration) rates during basal measurements. Plotted data represent mean and SD of 4 independent experiments. Bioenergetics analysis was performed by Seahorse technology (mitochondrial stress test).
E Mitochondrial electron transport chain (ETC) complexes were analyzed by Western blot. Values indicate the sum of all complexes normalized by vinculin as a loading control. Representative image of four independent experiments.
F Mitochondrial DNA copy number was assessed by quantitative PCR of mitochondrial-encoded genes and a nuclear-encoded gene using total DNA as template. Bars indicate the mean and error bars SEM of four independent experiments.
G Basal OCR/ECAR ratio. Plotted data represent mean and SD of four independent experiments. Bioenergetics analysis was performed by Seahorse technology (mitochondrial stress test). *P*-value is calculated with an unpaired two-sided Student's *t*-test.

© 2018 The Authors

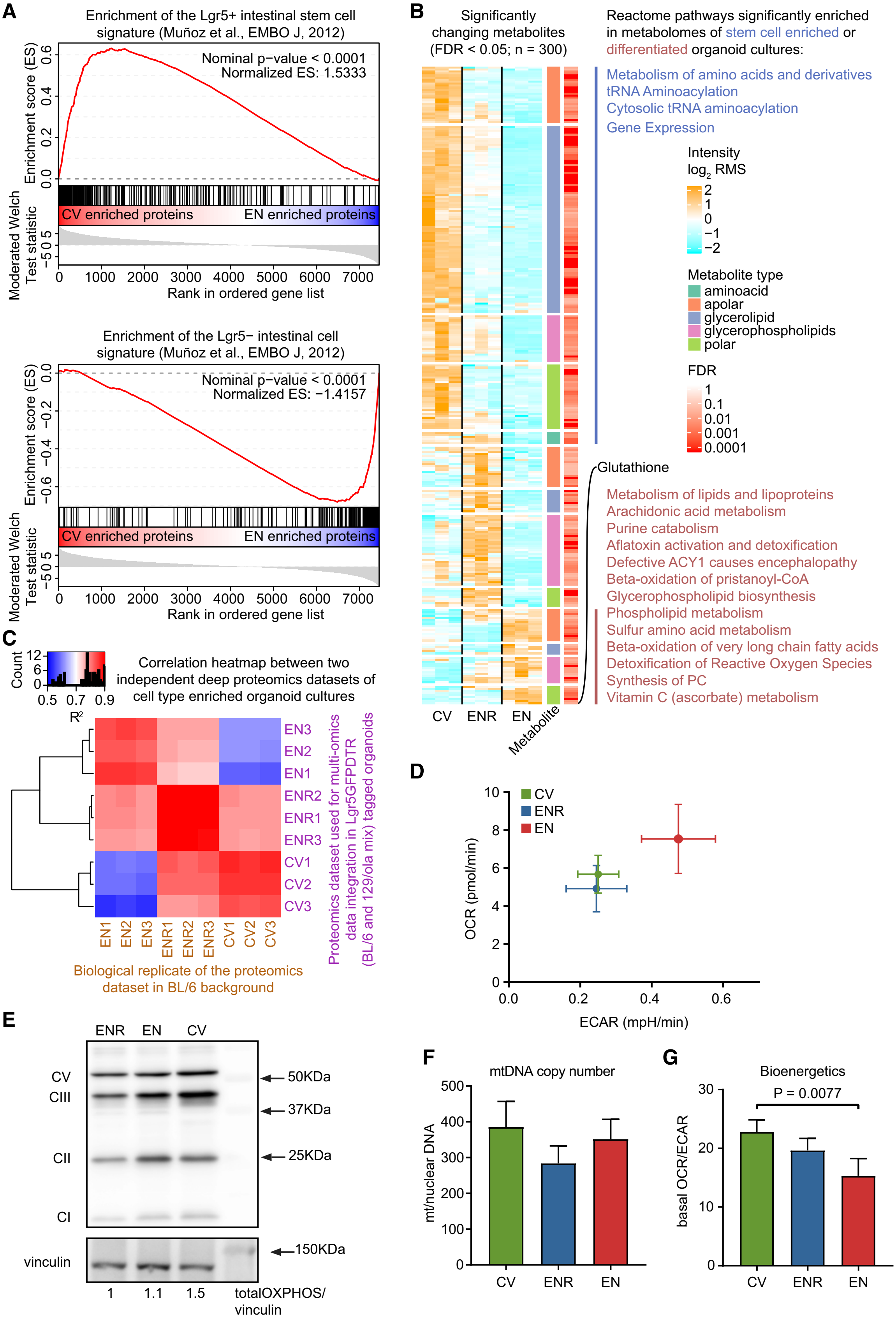

Figure EV1.

© 2018 The Authors

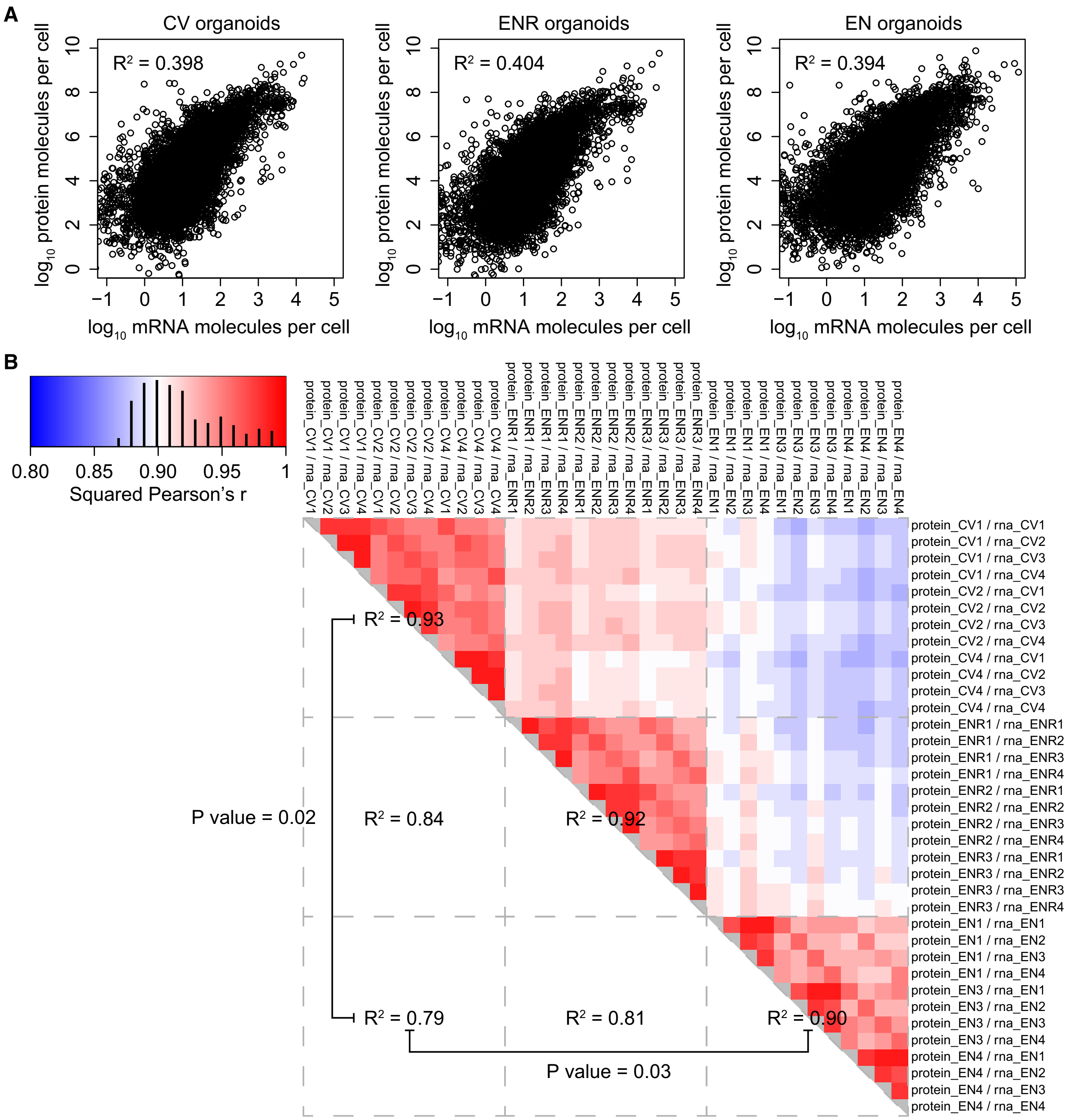

Figure EV2. Correlating protein to mRNA and proteins per mRNA ratios.

A Scatterplots showing the correlation between the proteome and transcriptome in the different organoid cultures.

B Correlation heatmap of the protein copies per mRNA molecule ratios for all different replicates. Test for significant difference between technical and biological variation was done with a *t*-test on the Fisher-transformed Pearson correlations.

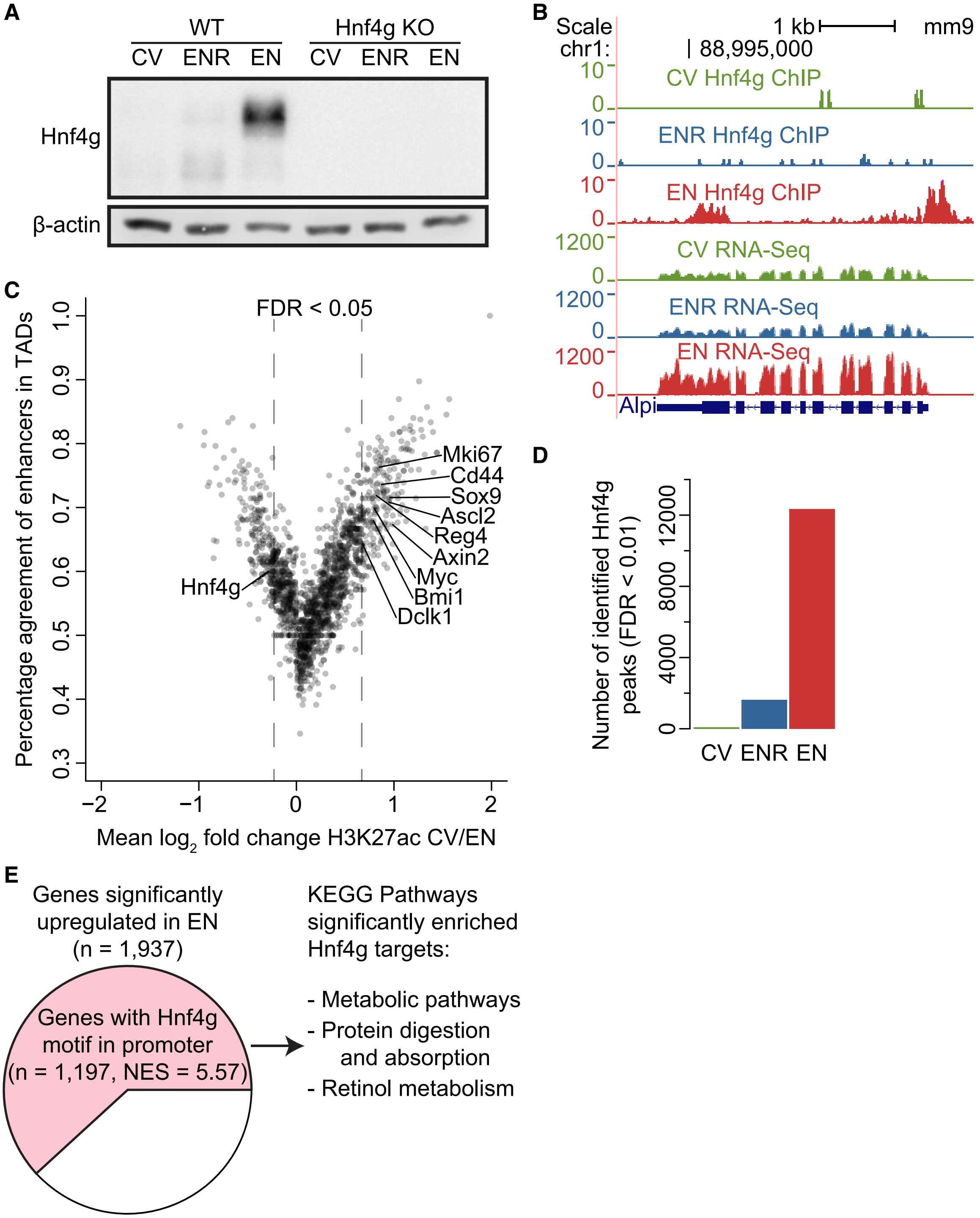

Figure EV3. Hnf4g dynamics and importance in the different cell-type-enriched organoids.

A Western blot for Hnf4g on whole cell extract of CV, ENR, and EN cultured WT and Hnf4g KO organoids. β-Actin is used as a loading control.
B ChIP-seq and RNA-seq profiles of CV, ENR, and EN WT organoids at the Alpi locus.
C Scatterplot showing the percentage of peaks inside a TAD that have fold change in H3K27ac signal in the same direction as the average fold change in H3K27ac of the whole TAD. Striped line demarcates the empirical 0.05 FDR cutoff. Significantly changing TADs that contain a known marker of intestinal homeostasis are highlighted.
D Number of identified peaks of the Hnf4g ChIP-seq in the different cell-type-enriched WT organoids.
E Pie chart showing all significantly upregulated genes in EN organoids. Genes with a Hnf4g motif in their promoter are highlighted in pink. Normalized enrichment score (NES) and motif association were determined in iRegulon (Janky *et al*, 2014). KEGG pathways that were significantly overrepresented (FDR < 0.01) in upregulated genes with a Hnf4g motif are listed.

 © 2018 The Authors

Figure EV4. Hnf4g importance in human colon cancer organoids and regulation of the Hnf4a locus.

A S-curve scatterplot with genes ranked by their average fold changes between STem cell Ascl2 Reporter (STAR) negative over STAR positive is plotted. HNF4G is highlighted in red. Average fold change is calculated from the colorectal tumor progression models and colorectal cancer patients reported in Oost *et al* (2018).

B Transcription factors ranked by their normalized enrichment score in the promoters of significantly downregulated genes ($P < 0.001$) in the STAR-positive samples from (A) were shown. Normalized enrichment score (NES) and motif association were determined in iRegulon (Janky *et al*, 2014).

C Genome browser windows over the complete Hnf4a locus showing all data and all used transcript databases.

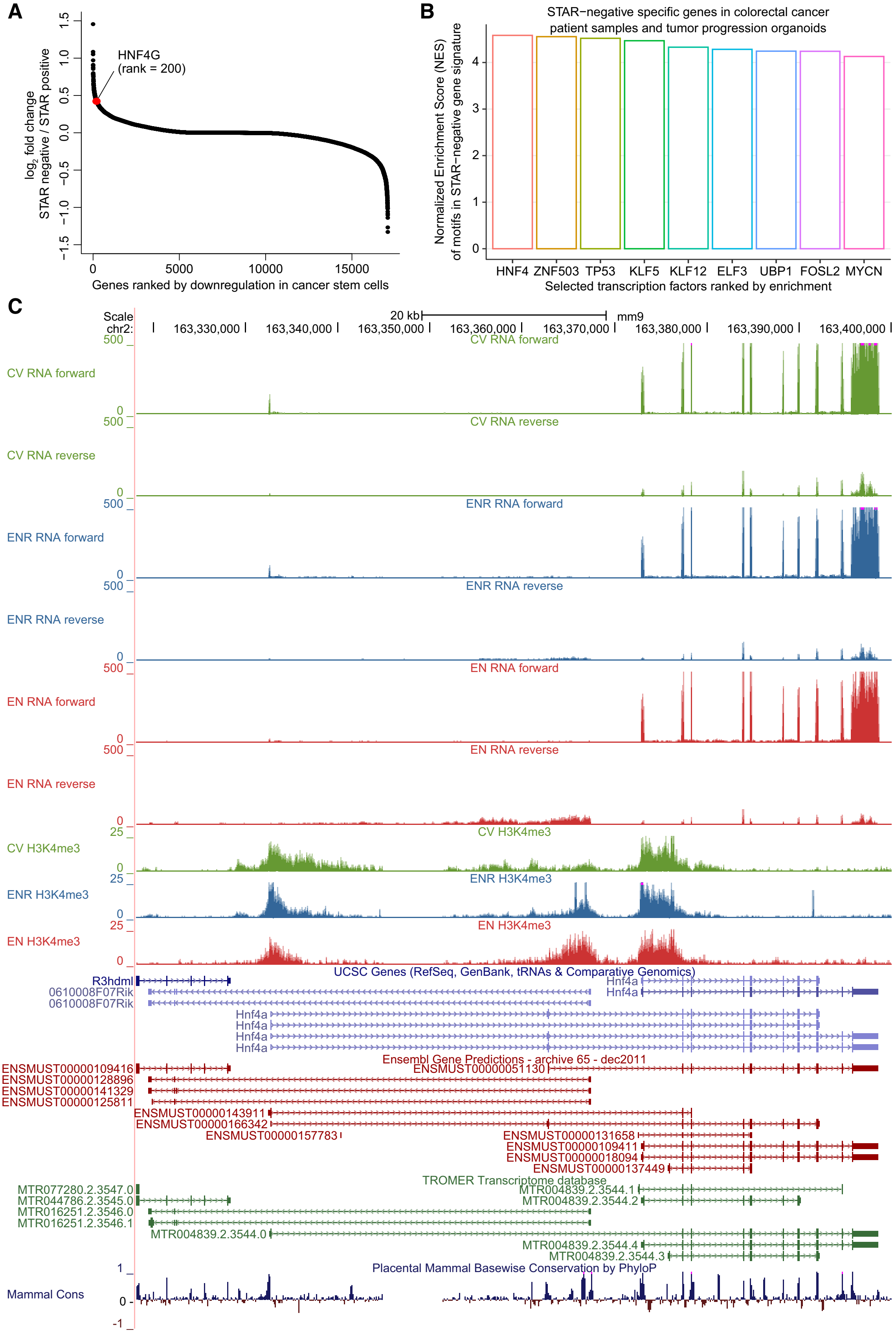

Figure EV4.

© 2018 The Authors

Figure EV5. Comparing histone profiles and DNA accessibility of secretory and enterocyte progenitor cells.

A Correlation heatmap comparing histone profiles and ATAC data from Jadhav *et al* (2017) to DNA accessibility and H3K27ac profiles of cell-type-enriched organoid cultures. Relative changes between CV and EN on significantly changing H3K27ac peaks were correlated to relative changes at the same sites between samples from each dataset of Jadhav *et al* (2017).

B Significantly changing sites between enterocyte and secretory progenitors were identified with the shown *P*-value threshold, and split in enterocyte or secretory-specific groups. Boxplot with the best HNF4 binding motif scores in enterocyte progenitor and secretory progenitor specific sites were plotted for each dataset. *P*-values comparing boxes are determined by two-tailed Mann–Whitney *U*-test. The central line in each boxplot represents the median, the notch around this line is the approximate 95% confidence interval, the hinges are the first and third quartile, and the whiskers extend to the lowest and highest values within 1.5× the interquartile range from the hinges.

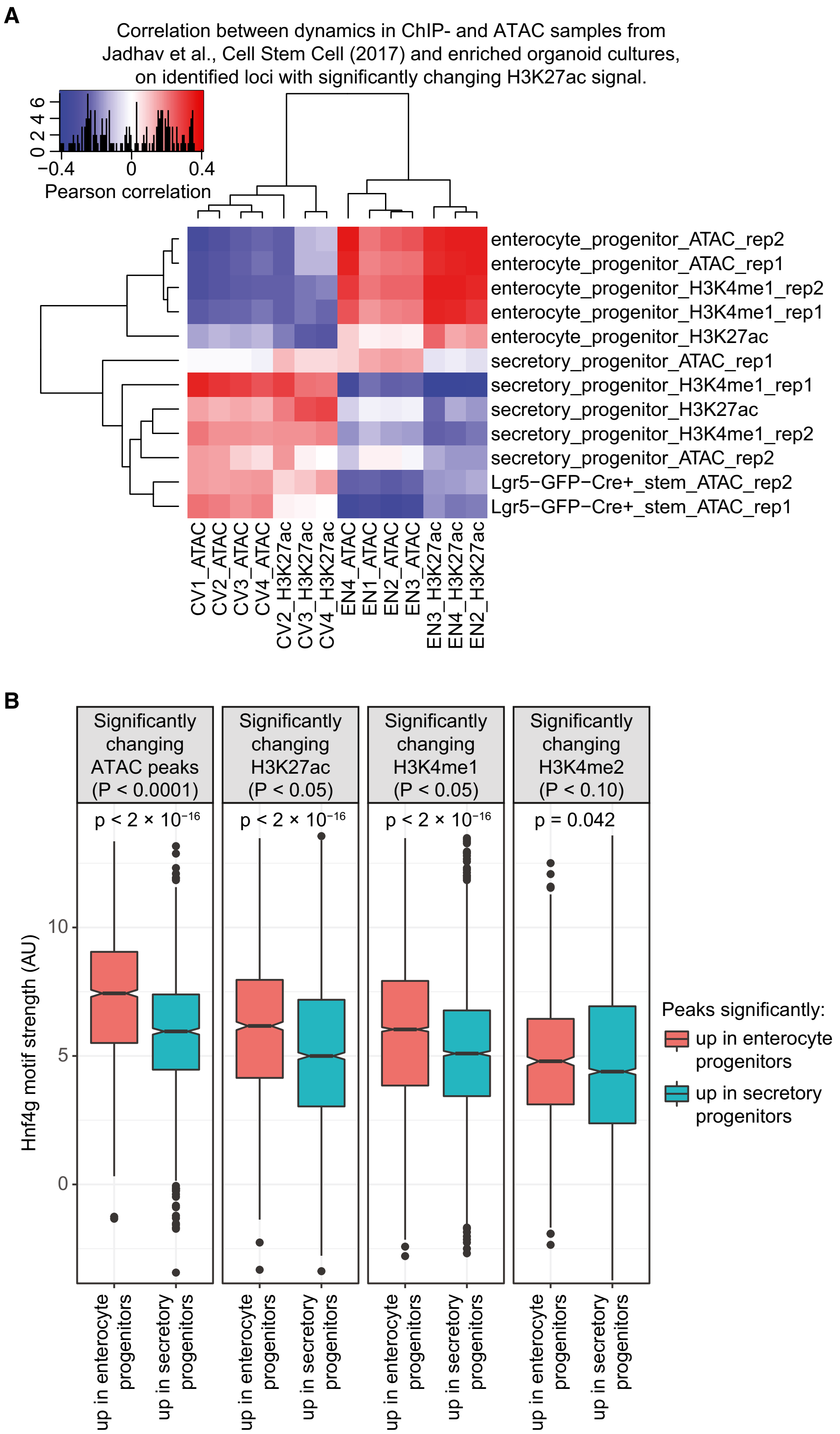

Figure EV5.

 © 2018 The Authors
